# Enhancing antioxidant delivery through 3D printing: a pathway to advanced therapeutic strategies

**DOI:** 10.3389/fbioe.2023.1256361

**Published:** 2023-10-04

**Authors:** Ageel Alogla

**Affiliations:** Industrial Engineering Department, College of Engineering (AlQunfudhah), Umm Al-Qura University, Mecca, Saudi Arabia

**Keywords:** 3D printing, antioxidants, oxidative stress-related disorders, therapeutic strategies, controlled release

## Abstract

The rapid advancement of 3D printing has transformed industries, including medicine and pharmaceuticals. Integrating antioxidants into 3D-printed structures offers promising therapeutic strategies for enhanced antioxidant delivery. This review explores the synergistic relationship between 3D printing and antioxidants, focusing on the design and fabrication of antioxidant-loaded constructs. Incorporating antioxidants into 3D-printed matrices enables controlled release and localized delivery, improving efficacy while minimizing side effects. Customization of physical and chemical properties allows tailoring of antioxidant release kinetics, distribution, and degradation profiles. Encapsulation techniques such as direct mixing, coating, and encapsulation are discussed. Material selection, printing parameters, and post-processing methods significantly influence antioxidant release kinetics and stability. Applications include wound healing, tissue regeneration, drug delivery, and personalized medicine. This comprehensive review aims to provide insights into 3D printing-assisted antioxidant delivery systems, facilitating advancements in medicine and improved patient outcomes for oxidative stress-related disorders.

## 1 Introduction

Oxidative stress, characterized by an imbalance between Reactive Oxygen Species (ROS) production and antioxidant defense mechanisms, has been implicated in the pathogenesis of numerous diseases, including cancer, neurodegenerative disorders, cardiovascular conditions, and inflammatory diseases (Charlton et al., 2020; Fundu et al., 2023; Z; Huang and Tan, 2023). Antioxidants play a vital role in mitigating the damaging effects of ROS and are widely recognized for their therapeutic potential (Poljsak, Šuput, and Milisav, 2013; Fairley et al., 2023). However, the efficient delivery of antioxidants to specific target sites within the body remains a significant challenge (Kang et al., 2018; Khalil et al., 2019).

In recent years, 3D Printing has emerged as a transformative technology with vast applications in various industries including aerospace (A.A. Alogla, Alzahrani, and Alghamdi, 2023), medicine and pharmaceuticals (Lin et al., 2019) and spare parts in general (A.A. Alogla et al., 2021; A; Alogla, Baumers, and Tuck, 2019). It enables the precise fabrication of complex three-dimensional structures with high accuracy, allowing for the customization of physical and chemical properties (Goyanes, Wang, et al., 2015; A; Alogla, 2021). This versatility has sparked growing interest in exploring the integration of antioxidants into 3D-printed constructs, aiming to enhance antioxidant delivery and improve therapeutic outcomes (Domínguez-Robles et al., 2019; Markovinović et al., 2023).

**FIGURE 1:**
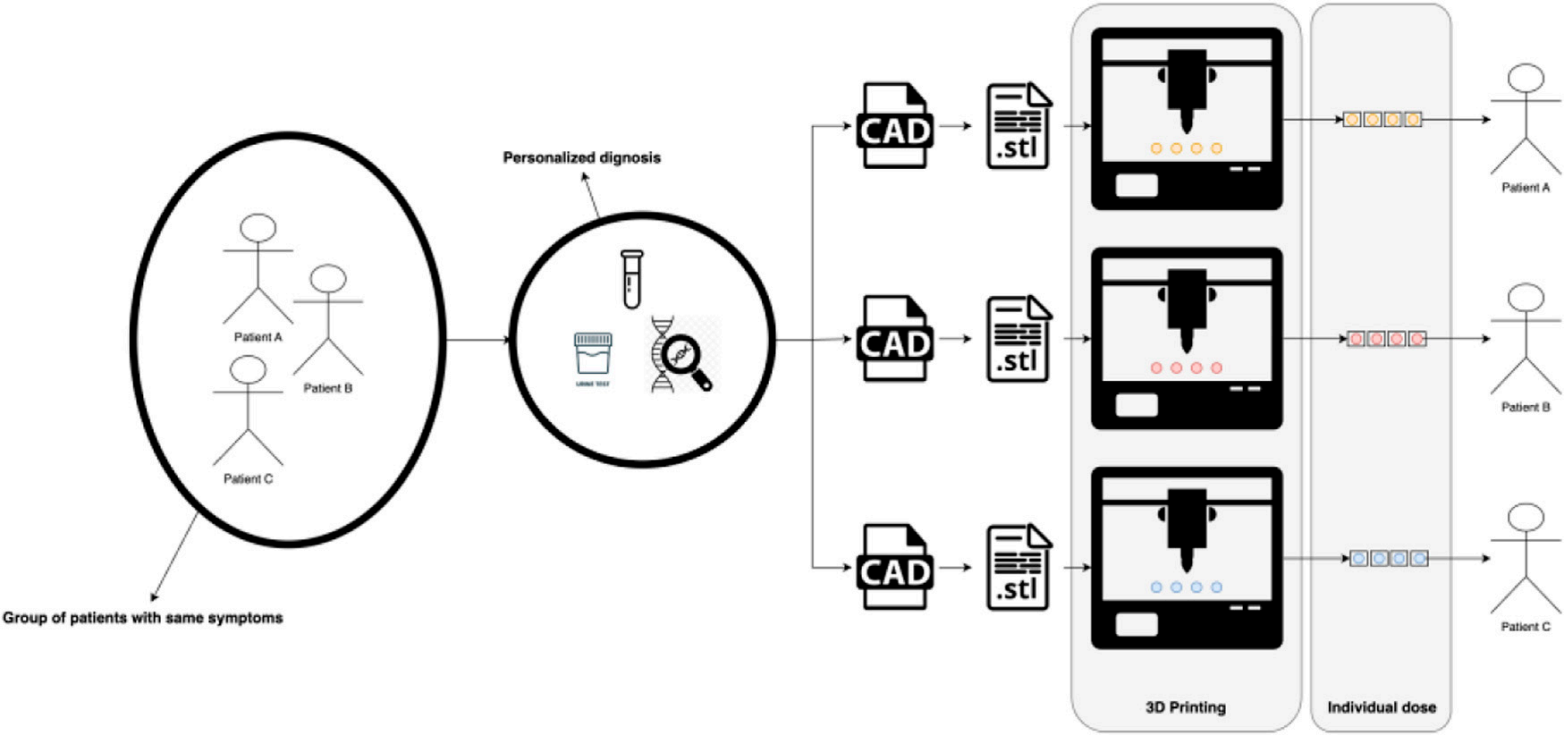
The use of 3D Printing in the context of personalized medicine.

Personalized medicine, a transformative paradigm in healthcare, finds a natural convergence with the innovative realm of 3D printing (Jain, 2002; Chan and Ginsburg, 2011; Vaz and Kumar 2021), see Figure 1. At the heart of personalized medicine lies the recognition that each patient is unique, necessitating tailored therapeutic approaches for optimal outcomes. This tailored approach encompasses not only genetic variations but also individualized anatomical intricacies. Herein, 3D printing emerges as a pivotal enabler, seamlessly fusing patient-specific data with the precision of fabrication (Serrano et al., 2023). By translating intricate medical imaging into tangible three-dimensional models, 3D Printing facilitates the creation of patient-specific implants, prosthetics, and drug delivery systems. This dynamic synergy between personalized medicine and 3D printing has the potential to revolutionize treatments, from orthopedic implants intricately customized to anatomical nuances, to drug-loaded structures meticulously designed for optimal release profiles based on individual patient needs. As personalized medicine continues to redefine healthcare, the versatility and precision of 3D printing stand poised to shape a future where therapies are uniquely tailored to each patient, ushering in an era of enhanced therapeutic efficacy and patient wellbeing.

The combination of 3D printing and antioxidants holds significant promise in the development of advanced therapeutic strategies (Hong, Purushothaman, and Song, 2020). By incorporating antioxidants within 3D-printed matrices, it becomes possible to achieve controlled release and localized delivery, overcoming limitations associated with traditional antioxidant administration routes (Zhang and Wang, 2022). Furthermore, the ability to tailor the release kinetics, spatial distribution, and degradation profiles of 3D-printed antioxidant systems offers opportunities for personalized medicine and individualized treatment approaches (Bagheri et al., 2023).

This review aims to provide a comprehensive overview of the synergistic relationship between 3D Printing and antioxidants in the context of therapeutic applications. This work will explore the current state of the art in 3D-printed antioxidant systems, discussing the various approaches employed for antioxidant encapsulation within 3D-printed constructs. Additionally, this work explores the influence of material selection, printing parameters, and post-processing methods on the release kinetics and stability of antioxidants. Furthermore, this work will highlight the potential applications of 3D-printed antioxidant systems across diverse fields, including wound healing, tissue regeneration, drug delivery, and personalized medicine. By addressing the challenges and opportunities associated with this emerging field, the aim of this paper is to inspire further research and development efforts towards the realization of advanced antioxidant delivery strategies.

## 2 Review methodology

In this review, a systematic approach was employed to comprehensively identify and analyze relevant literature pertaining to the integration of antioxidants into 3D-printed structures for enhanced therapeutic strategies. The systematic review process involved several sequential steps. First, to identify relevant studies, a systematic search of peer-reviewed literature across electronic databases, including PubMed, Google Scholar, Scopus, and Web of Science was conducted. The search strategy incorporated a combination of keywords and Medical Subject Headings (MeSH) terms, such as “3D printing,” “3D bioprinting,” “antioxidants,” “drug delivery systems,” “controlled release,” “encapsulation,” and “tissue regeneration.” The search strategy was tailored to each database, and the time frame of the search was limited to studies published from 2000.

The retrieved articles were screened then based on predefined inclusion and exclusion criteria. Inclusion criteria encompassed studies that focused on the incorporation of antioxidants into 3D-printed matrices, investigated the controlled release of antioxidants, and explored applications for drug delivery. Studies involving other encapsulation methods or non-3D printing techniques were excluded. The extracted data included study characteristics (e.g., authors, publication year), study design, antioxidant types, 3D printing materials and methods, encapsulation techniques, and applications, antioxidant release kinetics, stability, and *in vitro*/*in vivo* evaluations.

A narrative synthesis approach was employed to summarize the findings from the included studies. The results were organized based on the antioxidant types, 3D printing techniques, encapsulation methods, and therapeutic applications. This work aimed to present a coherent overview of the current state of research in 3D printing-assisted antioxidant delivery systems. It is acknowledged that a potential bias in this review process might occur, including publication bias and the possibility of missing unpublished studies. Additionally, limitations may arise from the variability in study designs and methodologies across the included studies.

## 3 Antioxidant delivery through 3D printing: components

To fabricate 3D-printed antioxidant delivery systems, a combination of materials is required for both the printing process and the encapsulation of antioxidants. This section provides a detailed description of the materials utilized in the process of 3D-printed antioxidants.

### 3.1 Printing materials

Biocompatible polymers such as polylactic acid (PLA), polycaprolactone (PCL), polyethylene glycol (PEG), and poly(lactic-co-glycolic acid) (PLGA) are commonly used in 3D printing (Pae et al., 2019; Chen et al., 2020; Liu et al., 2020; Brounstein, Yeager, and Labouriau, 2021). These materials offer good printability, mechanical strength, and biodegradability, making them suitable for biomedical applications (Athukoralalage et al., 2019). Hydrogels composed of natural polymers (e.g., gelatin, alginate, hyaluronic acid) or synthetic polymers (e.g., polyethylene oxide, polyvinyl alcohol) are employed for their ability to mimic the extracellular matrix and promote cell adhesion and proliferation (Chaudhuri et al., 2016; Ji et al., 2023). Composite materials, which are combinations of polymers and ceramics (e.g., hydroxyapatite, tricalcium phosphate) or polymers and metals (e.g., titanium), are utilized to enhance the mechanical and biological properties of the printed structures (Misra et al., 2011).

### 3.2 Materials and biocompatibility considerations in 3D printed antioxidant systems

The advancement of 3D printed antioxidant systems necessitates an astute selection of materials, encompassing antioxidants, solvents and carriers, and additives and stabilizers, each playing a vital role in the system’s overall effectiveness and biocompatibility.

Naturally occurring antioxidants like vitamins C and E, resveratrol, curcumin, and quercetin stand out due to their potent antioxidant properties and safety profiles (Esposito et al., 2019). However, their interactions with other 3D printing materials may impact their bioavailability or efficacy. On the other hand, synthetic antioxidants such as BHT and BHA offer stability but raise concerns about their long-term biocompatibility. When encapsulating these antioxidants, be it natural or synthetic, the choice of encapsulation materials, such as polymeric microspheres or nanoparticles, requires rigorous evaluation to ensure optimal delivery without compromising the antioxidant’s functionality (Hu et al., 2013).

The selection of solvents and carriers is pivotal in determining the biocompatibility of the printed construct. Solvents like ethanol, acetone, and DMSO can alter the biological integrity of printed constructs, and their implications should be weighed carefully (Costa et al., 2015; Zhou et al., 2018). Carriers such as emulsions, liposomes, and micelles can bridge the gap between hydrophobic antioxidants and hydrophilic printing materials but their interaction with biological environments, stability, and release kinetics merit in-depth scrutiny (Sommer et al., 2017).

Lastly, the use of additives and stabilizers cannot be understated in the 3D printing process. While plasticizers like PEG, glycerol, and triethyl citrate enhance the printability and flexibility of materials (J.H. Cho et al., 2008; Zhou et al., 2020; Jiang et al., 2020), their biocompatibility is of the essence. Similarly, stabilizers, which enhance the longevity and integrity of the printed constructs, must be chosen with caution, ensuring they do not leach and adversely interact with the surrounding environment (W. Liu et al., 2019; Johnson et al., 2023).

To encapsulate, the potential of 3D printing in antioxidant delivery is vast, yet every material and compound used in the process requires thorough vetting to ensure optimal biocompatibility, efficacy, and compliance with regulatory norms.

### 3.3 3D printing techniques employed

The fundamental concept of 3D Printing is the sequential layering of material to construct objects based on a 3D CAD design. As each layer is added, the resulting part gradually approximates the intended 3D CAD design. Thinner layers lead to a closer resemblance between the created part and the CAD model. All 3D Printing technologies share the layer-by-layer approach to building parts, but they vary in terms of compatible materials, layering mechanisms, and fusion methods. These distinctions contribute to variations in build time, post-processing requirements, and costs across different 3D Printing techniques. Moreover, these differences determine the necessary accuracy level for the target part and its corresponding material and mechanical properties.

**FIGURE 2:**
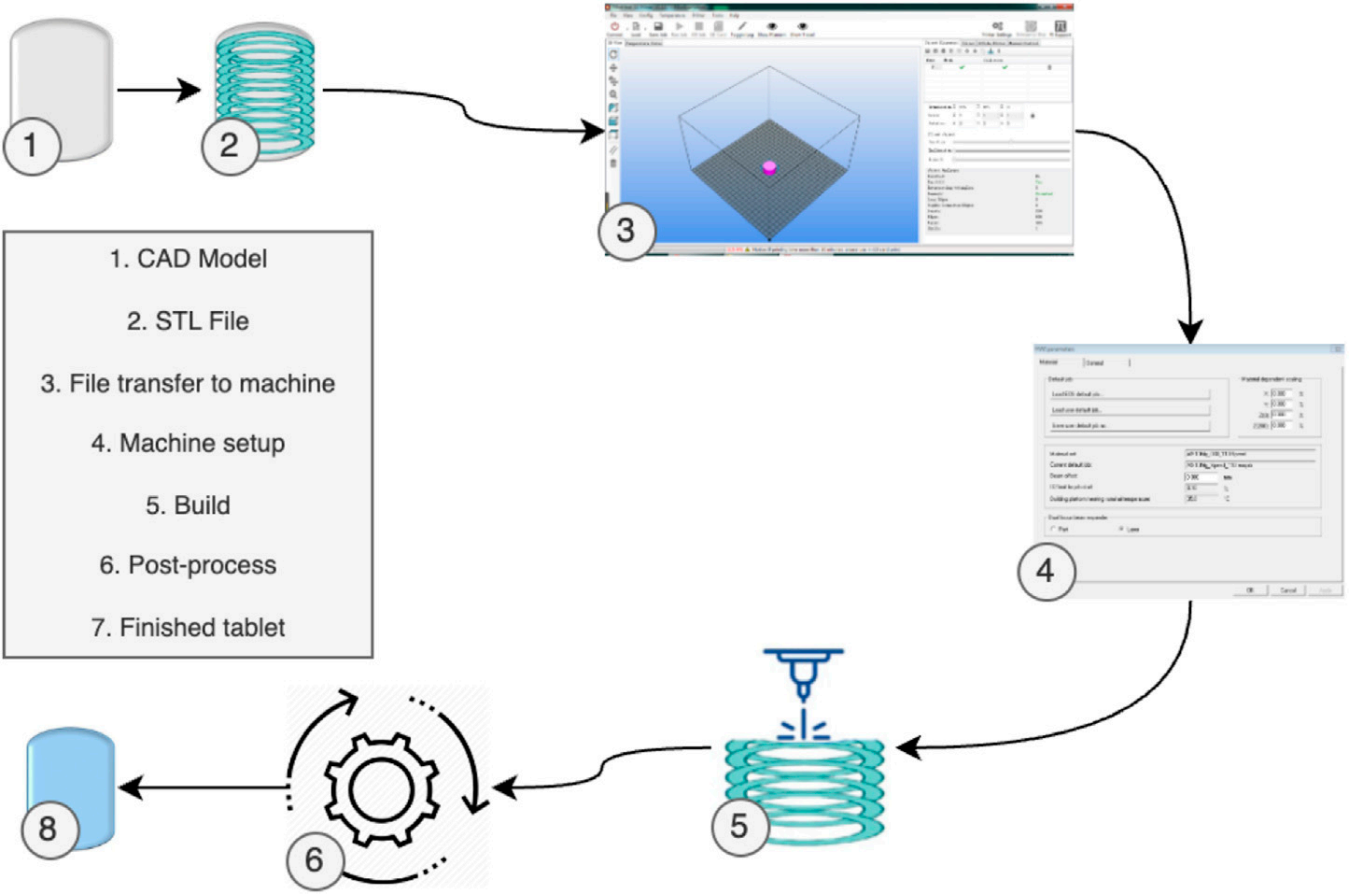
3D Printing generic process of pharmaceuticals [adapted from (Pavan Kalyan and Kumar 2022) with permission].

Despite these variations, most 3D Printing processes generally follow a standard procedure (Mueller, 2012), as depicted in Figure 2. Initially, the part design is created using CAD software. Subsequently, the CAD model is converted into an STL file, allowing the AM machine to recognize it. In the third step, the STL file is transferred to the 3D Printing machine, with any necessary adjustments made regarding size, position, or orientation. Following this, machine setup occurs in the fourth step, involving calibration of build parameters like layer thickness and cooling duration. The actual build process, involving layer deposition, takes place as the fifth step. The sixth step encompasses removing the printed part from the 3D Printing machine. Post-processing activities are conducted in the seventh step to refine the part. Ultimately, the fully processed part is prepared for its intended use.

Various 3D printing techniques have been employed for the fabrication of drug delivery and antioxidant-loaded structures. Each technique offers unique advantages in terms of resolution, speed, material compatibility, and complexity of geometries. This section provides an overview of the 3D printing techniques commonly used in the context of antioxidant delivery systems.

1. Fused Deposition Modeling (FDM): FDM, also known as fused filament fabrication, is one of the most widely used 3D printing techniques. In FDM, a thermoplastic filament is melted and extruded through a nozzle. Layer-by-layer, the molten filament is deposited onto a build platform, gradually forming the desired structure. FDM allows for rapid prototyping and offers a wide range of compatible printing materials, including polymers and composite materials. With FDM, antioxidants can be infused into the thermoplastic filament during the manufacturing process. For example, antioxidants such as vitamin E can be blended with PLA (polylactic acid) filaments. This approach can result in a filament that offers antioxidant properties upon degradation of the printed part (Katsiotis et al., 2021).
2. Stereolithography (SLA) and Digital Light Processing (DLP): SLA and DLP utilize similar principles of photopolymerization. In SLA, a liquid resin is exposed to a specific wavelength of light, typically ultraviolet (UV), which solidifies the resin layer by layer to create the final 3D-printed structure. DLP follows a similar process but uses a digital light projector to project UV light patterns onto the resin, solidifying it layer by layer. Both techniques offer high resolution and the ability to fabricate intricate structures. Photocurable resins used in SLA and DLP can be tailored to contain antioxidants. For instance, quercetin, a plant flavonoid with antioxidant properties, can be mixed with the resin. The final cured structure can release the antioxidant over time upon degradation (Healy et al., 2019).
3. Selective Laser Sintering (SLS): SLS involves the use of a high-powered laser to selectively fuse powdered materials, typically polymers or metals, layer by layer. The laser selectively melts the powder particles, creating solidified layers that gradually form the desired 3D structure. SLS offers excellent material compatibility, allowing for the use of a wide range of polymers and composites. Powders used in SLS can be pre-mixed with antioxidants. Curcumin-loaded polymer powders can be used in SLS to obtain 3D structures with inherent antioxidant properties, releasing curcumin upon degradation or interaction with a specific stimulus (Zhang et al., 2023).
4. Inkjet-based Printing: Inkjet-based printing employs printheads that deposit droplets of liquid materials onto a substrate or build platform. The liquid materials, which can include polymers, hydrogels, or a combination of both, form successive layers to create the final 3D structure. Antioxidants can be included in the liquid inkjet ink, enabling their incorporation into the printed structures. Inkjet-based printing offers high resolution, compatibility with a variety of materials, and the potential for precise placement of antioxidants within the printed constructs. An example might be an alginate-based hydrogel ink containing resveratrol, enabling the antioxidant to be integrated within the printed structure and released upon degradation.
5. Multi-Jet Printing (MJP) is an additive manufacturing technology that falls under the category of 3D printing. It utilizes a similar layer-by-layer approach to other 3D printing methods, but it distinguishes itself by using multiple inkjet printheads to simultaneously deposit and solidify layers of liquid photopolymer material. In MJP, the process involves creating a three-dimensional object by selectively jetting and curing layers of liquid photopolymer material with UV light. This is done using an array of printheads that can deposit tiny droplets of the photopolymer material onto the build platform. Each printhead jets the material in a precise pattern based on the digital design, and the material solidifies when exposed to UV light. The photopolymer materials used in MJP can be developed to incorporate antioxidants. Resins containing ascorbic acid (vitamin C) can be utilized in MJP to obtain structures with antioxidant properties, ensuring controlled release over time (Mieloszyk et al., 2020).
6. Binder Jetting (BJ) Binder Jetting is an additive manufacturing technology used for creating three-dimensional objects layer by layer. It falls under the category of 3D printing and is known for its speed, cost-effectiveness, and ability to produce complex geometries. In Binder Jetting, the process involves depositing a powdered material layer by layer and selectively applying a liquid binding agent to solidify the powder and form the desired object. Binders used in BJ can be designed to contain antioxidants. The binder can be mixed with antioxidants like vitamin E, enabling the printed parts to showcase antioxidant characteristics upon interaction with the environment or specific triggers (Ziaee and Crane, 2019).
7. Pneumatic Extrusion (PE), also known as air-driven extrusion, is a process used in various industries, including additive manufacturing (3D printing), food processing, and materials fabrication. It involves using compressed air or gas pressure to push a material, typically a viscous or semi-solid substance, through a nozzle or an aperture to create a desired shape or structure. The material used in PE can be pre-mixed with antioxidants before extrusion. For instance, a collagen-based material can be mixed with antioxidants like green tea extracts, ensuring the antioxidant is embedded within the extruded structure (H.-W. Cho et al., 2020).

It is important to select a suitable 3D printing technique based on the desired properties of the antioxidant delivery system, including resolution, material compatibility, scalability, and the ability to achieve controlled release. The choice of technique will depend on the specific requirements of the application, the materials used, and the available resources.

Table 1 provides a comprehensive overview of the major biomaterials employed in various AM technologies. The integration of biomaterials with AM techniques has opened new avenues for advancing tissue engineering, drug delivery, and regenerative medicine. This table highlights the diverse range of biomaterials utilized in conjunction with specific AM methods, each tailored to leverage the unique capabilities of the respective technology. The biomaterials encompass a variety of polymers, hydrogels, ceramics, and composites, among others, each selected for their compatibility with the AM process and their potential to enhance therapeutic outcomes.

**TABLE 1:** Major biomaterials utilized for each 3D Printing technology with the relevant literature.

3D printing technology	Biomaterial	References
Multi-Jet Printing (MJP)	PEGDA (Poly(ethylene–glycol) diacrylate)	(Acosta-Vélez et al., 2018)
	HPC (Hydroxypropyl cellulose)	(Kollamaram et al., 2018)
	PLGA (poly(lactic-co- glycolic acid))	(Gu et al., 2012)
Stereolithography (SLA)	PEGDA (Poly(ethylene–glycol) diacrylate)	(Martinez et al., 2018)
Digital Light Processing (DLP)	PEGDA (Poly(ethylene–glycol) diacrylate)	Kadry et al., 2019; Krkobabić et al., 2019; Li et al., 2020
Fused Deposition Modelling (FDM)	PVP (Polyvinylpyrrolidone)	(Okwuosa et al., 2016)
	HPC (Hydroxypropyl cellulose)	(Melocchi et al., 2015)
	Eudragit	(Goyanes, Chang, et al., 2015)
	PU (Polyurethane)	(Hung et al., 2016)
	EC (Ethyl cellulose)	(Yang et al., 2018)
	PLGA (poly(lactic-co- glycolic acid))	(Zhou, Yao, et al., 2018)
	TEC (Triethyl Citrate)	(Arafat et al., 2018)
	PEO (Polyethylene Oxide)	(Isreb et al., 2019)
	TPU (Thermoplastic polyurethane)	(Mathew et al., 2019)
Pneumatic Extrusion (PE)	PVP (Polyvinylpyrrolidone)	Okwuosa et al., 2017; Cui et al., 2019
	HPC (Hydroxypropyl cellulose)	Goyanes et al., 2019; Ong et al., 2020
	PLGA (poly(lactic-co-glycolic acid))	(Yi et al., 2016)
	Gelatin	Shim et al., 2014; Lee et al., 2016
	TPU (Thermoplastic polyurethane)	(Welsh et al., 2019)
Binder Jetting (BJ)	PVP (Polyvinylpyrrolidone)	(Katstra et al., 2000)
	HPC (Hydroxypropyl cellulose)	(Thabet, Lunter, and Breitkreutz, 2018)
	Eudragit	(Katstra et al., 2000; Rowe et al., 2000)
	EC (Ethyl cellulose)	(Yu et al., 2007; Yu, Branford-White, et al., 2009; Yu, Shen, et al., 2009)
	Tween20	(Katstra et al., 2000)
Selective Laser Sintering (SLS)	Eudragit	(Fina et al., 2017; Fina, Goyanes, et al., 2018; Fina, Madla, et al., 2018)
	EC (Ethyl cellulose)	(Awad et al., 2019)
	PEO (Polyethylene Oxide)	(Trenfield et al., 2020)

### 3.4 Antioxidant selection and characterization methods

The selection and characterization of antioxidants are crucial steps in the development of 3D printing-assisted antioxidant delivery systems. This section discusses the considerations for antioxidant selection and highlights the characterization methods commonly employed to evaluate their properties. The choice of antioxidants depends on several factors, including their potency, stability, compatibility with printing materials, and therapeutic relevance. Natural antioxidants, such as vitamins C and E, resveratrol, curcumin, and quercetin, are often preferred due to their well-documented antioxidant properties and potential health benefits (Brewer, 2011; Kagathara et al., 2022). Synthetic antioxidants, such as BHT and BHA, are also considered for their stability and effectiveness in preventing oxidative damage (Hong, Purushothaman, and Song, 2020).

Factors to consider when selecting antioxidants include their solubility in the printing materials or carrier solutions, compatibility with the printing process, and their intended release kinetics (Hong, Purushothaman, and Song, 2020; Shahbazi et al., 2021). Antioxidants with different solubilities, hydrophobic or hydrophilic, may require specific encapsulation techniques or modifications to achieve controlled release profiles. Several characterization methods are employed to assess the properties of antioxidants and ensure their suitability for incorporation into 3D-printed structures (Vieira et al., 2020). The first of these is chemical characterization. Techniques such as nuclear magnetic resonance (NMR), high-performance liquid chromatography (HPLC), and gas chromatography-mass spectrometry (GC-MS) can be utilized to confirm the identity and purity of antioxidants. These methods help determine the presence of impurities or degradation products that could impact the antioxidant’s stability and efficacy.

Another characterization method is antioxidant activity assays. Various assays are available to evaluate the antioxidant activity of compounds. These include the 2,2-diphenyl-1-picrylhydrazyl (DPPH) assay, oxygen radical absorbance capacity (ORAC) assay, ferric reducing antioxidant power (FRAP) assay, and total phenolic content determination. These assays provide quantitative measurements of the antioxidant’s ability to scavenge free radicals and protect against oxidative damage. Additional characterization method is stability assessment, where stability studies are performed to evaluate the degradation kinetics and shelf-life of antioxidants. Factors such as temperature, humidity, light exposure, and pH can influence antioxidant stability. The stability of antioxidants is profoundly influenced by various external factors. Elevated temperatures can accelerate degradation reactions, as seen with vitamin C, which oxidizes rapidly under heat. Humidity can also compromise stability, with moisture facilitating the hydrolysis of compounds like the flavonoids in green tea extracts. Light exposure, particularly ultraviolet light, presents concerns for antioxidants such as beta-carotene, which undergoes photodegradation, leading to a loss in its vibrant color and antioxidant activity. Lastly, the pH level can play a pivotal role in determining antioxidant efficacy, with molecules like anthocyanins in berries undergoing structural and potency shifts with pH changes. Thus, when storing or formulating products containing antioxidants, it is essential to account for these factors to ensure their sustained integrity and efficacy. Accelerated stability tests, including accelerated aging and stress testing, are conducted to simulate long-term storage conditions and assess the antioxidant’s degradation behavior.

Compatibility testing is also an important characterization method. Compatibility between the antioxidant and printing materials or carrier solutions is essential to ensure a successful incorporation process. Compatibility tests may include assessing the antioxidant’s solubility in printing materials or carriers, evaluating potential interactions (e.g., chemical reactions or phase separation) between the antioxidant and the printing materials, and investigating the impact of the antioxidant on the mechanical or rheological properties of the printing materials. Final characterization method is the release kinetics evaluation. To assess the release kinetics of antioxidants from 3D-printed structures, methods such as UV-Vis spectrophotometry, HPLC, or fluorescence spectroscopy can be employed. These techniques enable the quantification of released antioxidants over time, providing insights into their release profiles and rates.

By employing these characterization methods, researchers can ensure the quality, stability, and effectiveness of the selected antioxidants for incorporation into 3D-printed antioxidant delivery systems. Such characterization ensures that the desired therapeutic properties and controlled release kinetics of antioxidants are achieved, thereby enhancing the overall efficacy of the developed systems.

## 4 3D printing-assisted antioxidant delivery systems

### 4.1 Direct mixing approach: incorporation of antioxidants within the printing materials

In 3D printing-assisted antioxidant delivery systems, one approach is to directly mix antioxidants within the printing materials prior to the printing process. This section discusses the direct mixing approach and its implications for the incorporation of antioxidants into the printing materials. The direct mixing approach involves the homogenous dispersion of antioxidants within the printing materials, such as polymers, hydrogels, or composite formulations (Alhijjaj, Belton, and Qi, 2016; Goyanes et al., 2019). This can be achieved through various methods, including physical blending, melt mixing, or solvent mixing. The goal is to uniformly distribute the antioxidants throughout the printing material matrix, enabling their controlled release upon degradation or post-printing modification.

**FIGURE 3:**
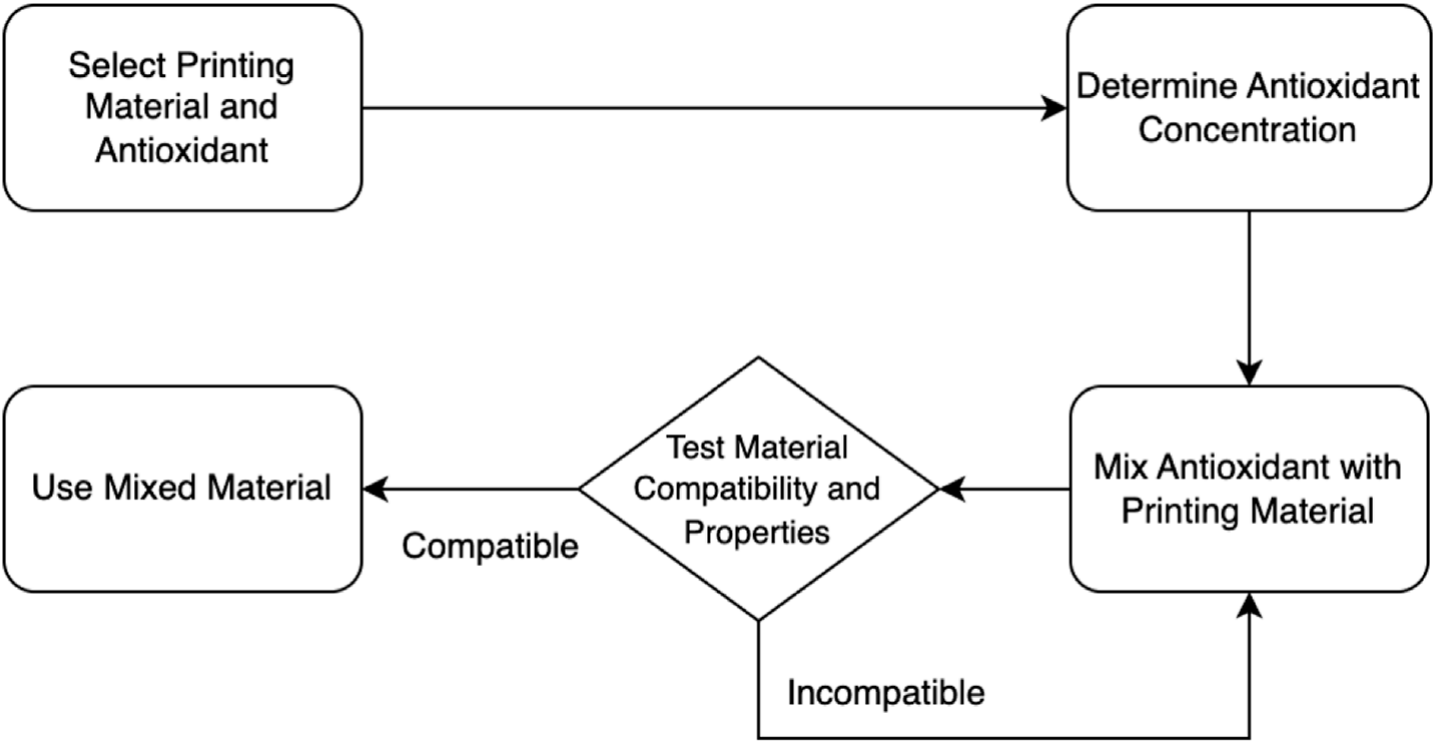
A flowchart of the direct mixing approach incorporation of antioxidants within the printing materials.

In Figure 3, the direct mixing approach for incorporating antioxidants within the printing materials is outlined. It starts with the selection of the printing material and antioxidant. The concentration of the antioxidant is then determined. Next, the antioxidant is mixed with the printing material. The resulting mixed material undergoes testing for compatibility and properties. Based on the test results, a decision point is reached. If the material is deemed compatible and possesses the desired properties, it can be used for printing. Otherwise, if it is found incompatible or lacks the desired properties, further adjustments or considerations may be necessary.

One advantage of the direct mixing approach is its simplicity and ease of implementation. By incorporating antioxidants directly into the printing materials, the need for additional encapsulation steps or modification of the printing process is eliminated, simplifying the overall fabrication process. This approach also allows for a higher loading capacity of antioxidants within the printing materials, providing the potential for sustained and prolonged release. However, the direct mixing approach presents challenges and considerations that need to be addressed, including:

1. Material compatibility: The compatibility between the antioxidant and the printing material is crucial to ensure a uniform dispersion and prevent phase separation. The selection of printing materials should take into account the solubility and compatibility of the chosen antioxidants to achieve a stable blend.
2. Antioxidant stability: The printing process, including heat exposure during melt-based techniques, may subject antioxidants to thermal degradation or chemical reactions that can reduce their stability and efficacy. Therefore, it is essential to select antioxidants that are resistant to the printing conditions or incorporate stabilizers to maintain their antioxidant activity.
3. Release kinetics control: Directly mixed antioxidants may exhibit burst release or uncontrolled release profiles due to their immediate exposure to the surrounding environment upon degradation of the printing material. The release kinetics of the antioxidants can be influenced by the degradation rate and porosity of the printed structure. Strategies such as adjusting the printing parameters, incorporating controlled degradation modifiers, or using gradient structures can be employed to achieve desired release profiles.
4. Optimization of antioxidant loading: The concentration of antioxidants within the printing materials should be carefully optimized to achieve the desired therapeutic effect without compromising the mechanical properties or processability of the printed constructs. Excessive loading of antioxidants may lead to material degradation, reduced printability, or altered mechanical properties.

The direct mixing approach has been utilized in various applications, including wound healing, tissue engineering, and drug delivery (Ruffert et al., 2006; Shaqour et al., 2020). By incorporating antioxidants directly within the printing materials, it allows for their controlled release at the site of interest, providing localized antioxidant therapy and potentially enhancing treatment outcomes.

### 4.2 Coating techniques: surface modification and encapsulation of antioxidants onto printed structures

Surface modification in 3D printing-assisted antioxidant delivery systems involves the application of a thin layer of antioxidant-containing material onto the surface of the printed structures. Techniques such as dip coating, spray coating, or electrostatic deposition are utilized to achieve this surface modification (Shi et al., 2007). The purpose of surface modification is to create a protective barrier that separates the printed material from the surrounding environment while allowing for controlled release of antioxidants.

**FIGURE 4:**
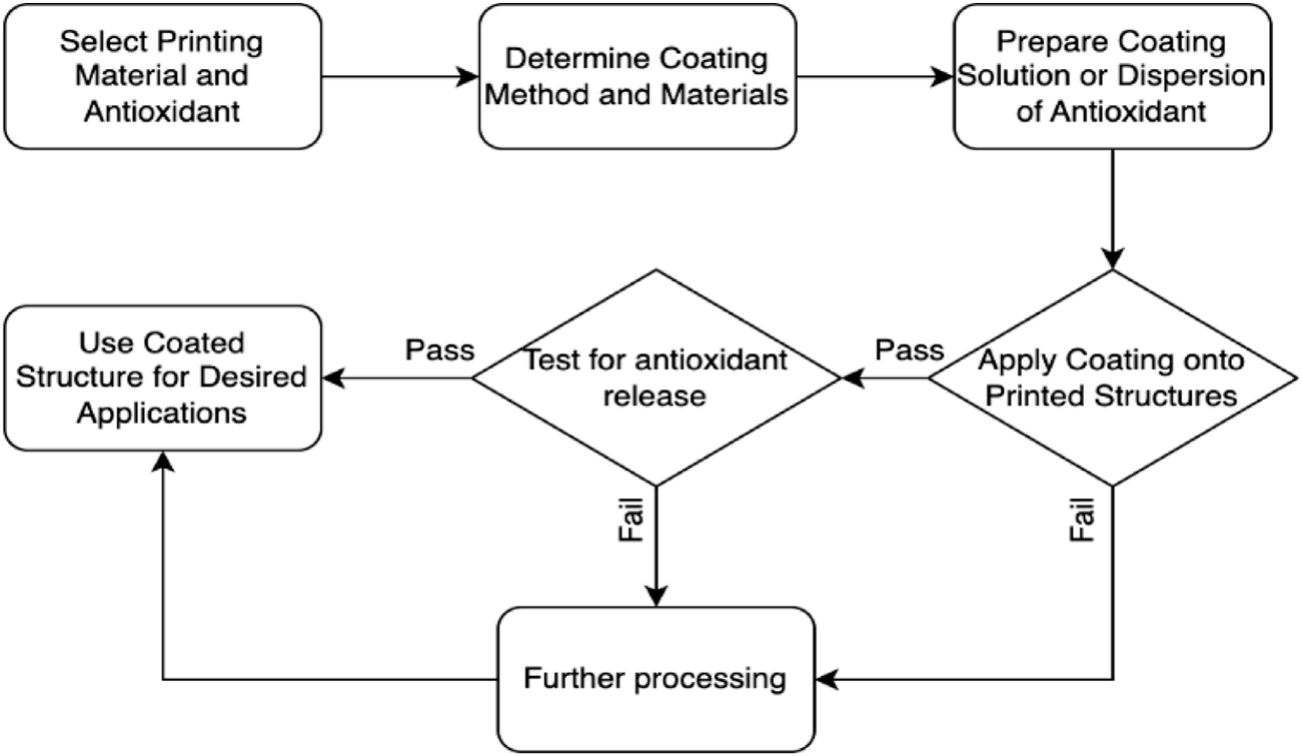
A flowchart of surface modification and encapsulation of antioxidants onto printed structures.

In Figure 4, the coating techniques for surface modification and encapsulation of antioxidants onto printed structures are illustrated. The process starts with the selection of the printing material and antioxidant. The appropriate coating method and materials are determined. A coating solution or dispersion of the antioxidant is prepared. The coating is then applied onto the printed structures. The quality and adhesion of the coating are assessed. Based on the assessment, a decision point is reached. If the coating quality and adhesion are satisfactory, the coated structure can be used for the desired applications. If not, further adjustments or considerations may be necessary. Additionally, the coated structure can also be tested for antioxidant release to ensure the desired properties and functionality are achieved. One significant advantage of surface modification is the ability to achieve controlled release kinetics. The coating layer acts as a reservoir for the antioxidants, gradually releasing them over time. This controlled release mechanism ensures a sustained and prolonged delivery of antioxidants, which can be particularly beneficial in applications such as wound healing or tissue regeneration where a continuous supply of antioxidants is desired.

The surface modification technique also offers versatility in terms of the choice of coating materials. Various biocompatible polymers, hydrogels, or lipid-based materials can be employed to encapsulate antioxidants. The selection of coating materials depends on factors such as the desired release profile, compatibility with the printing material, and the targeted application. The coating layer can be tailored to accommodate specific antioxidant properties and optimize their functionality. In addition to controlled release, surface modification can provide additional functionalities to the printed structures. For instance, the coating layer can offer protection against oxidation, UV radiation, or microbial contamination, safeguarding both the printed material and the encapsulated antioxidants. Furthermore, the coating layer can improve the biocompatibility of the printed structures, promoting cell adhesion, proliferation, and tissue integration.

It is worth noting that surface modification techniques may require careful optimization to ensure uniform coating distribution and adequate adhesion to the printed structures. Factors such as coating thickness, deposition method, and curing parameters need to be considered to achieve consistent and reproducible coating results. Characterization methods, including imaging techniques and release kinetics evaluation, can be employed to assess the uniformity, stability, and release behavior of the surface-modified structures.

### 4.3 Encapsulation methods: strategies for embedding antioxidants within 3D-Printed matrices

Encapsulation methods offer an alternative approach for incorporating antioxidants within 3D-printed structures, providing enhanced protection and controlled release. This section discusses various encapsulation strategies used to embed antioxidants within the matrices of 3D-printed constructs. Encapsulation techniques involve the entrapment of antioxidants within a protective matrix material, forming a reservoir that can be distributed throughout the printed structure. These methods provide several advantages, including controlled release, improved stability, and the ability to target specific areas of interest within the printed constructs.

One commonly employed encapsulation method is the use of microspheres or nanoparticles loaded with antioxidants (Warsi et al., 2018; Wang et al., 2019). These small particles are fabricated using techniques such as emulsion, solvent evaporation, or coacervation, and can be incorporated into the printing materials or deposited onto the printed structures post-printing. The encapsulated antioxidants are gradually released from the microspheres or nanoparticles, providing a sustained and controlled release profile. In the realm of antioxidant encapsulation, various materials have been employed to formulate particles that optimize delivery. For instance, the biodegradable polymer PLGA is favored for its biocompatibility and adjustable degradation rates, ideal for both hydrophobic and hydrophilic antioxidants. Natural biopolymer chitosan, with its unique mucoadhesive properties, aids in enhancing the stability and bioavailability of antioxidants, while liposomes, made of phospholipid vesicles, protect and deliver both water and fat-soluble antioxidants. Notably, hydrophobic polyphenol curcumin often finds itself encapsulated in nanoparticles or liposomes to tackle its solubility and stability issues. Meanwhile, the water-soluble Vitamin C, vulnerable to oxidation, benefits from encapsulation in chitosan nanoparticles or liposomes. Another antioxidant, resveratrol, also uses similar encapsulation strategies to boost its bioavailability. The release profiles of these encapsulated antioxidants vary, with some showing a burst release pattern, especially in liposomes, where a large fraction is rapidly discharged initially. However, more consistent, sustained releases are common with PLGA nanoparticles, while controlled release can be engineered using techniques like coacervation or layer-by-layer assembly, allowing precise modulation of release rates.

Another approach is the encapsulation of antioxidants within hydrogel-based matrices (Fedorovich et al., 2009; Erünsal and Emir, 2022; Züger et al., 2022). Hydrogels, which are crosslinked networks of hydrophilic polymers, offer a three-dimensional structure capable of retaining a large amount of water and encapsulated molecules. Antioxidants can be loaded into the hydrogel matrix during or after the printing process, enabling their controlled release over time. Hydrogel-based encapsulation provides a biocompatible environment and can be designed to respond to external stimuli, such as pH or temperature, further modulating the release kinetics of antioxidants.

In addition to microspheres, nanoparticles, and hydrogels, other encapsulation methods include liposomes (Liu et al., 2020), polymeric films or coatings (Germini and Peltonen, 2021), and electrospun fibers (Kendal et al., 2017). Liposomes are lipid-based vesicles that can entrap antioxidants within their bilayer structure, offering controlled release properties. Polymeric films or coatings can be applied to the printed structures, serving as a protective layer and reservoir for antioxidants. Electrospun fibers, produced by electrostatically spinning polymer solutions or melts, can incorporate antioxidants within their fibrous structure, allowing for controlled release and mimicking the extracellular matrix.

The choice of encapsulation method depends on various factors, including the desired release kinetics, compatibility with the printing materials, and the targeted application. Each method offers unique advantages in terms of the encapsulation efficiency, stability, and control over release profiles. Optimization of encapsulation parameters, such as particle size, polymer composition, and loading efficiency, is crucial to achieve the desired therapeutic effect and ensure the integrity of the printed structures. Characterization techniques, such as microscopy, spectroscopy, and release kinetics evaluation, are employed to assess the encapsulation efficiency, distribution of antioxidants within the printed structures, and the release behavior. Furthermore, the biocompatibility and potential cytotoxicity of the encapsulated antioxidants should be carefully evaluated to ensure their safety for biomedical applications. Table 2 provides a simple yet complete overview of how different 3D printing methods work together with various antioxidants loading methods. This table helps understand the appropriate combinations of techniques for putting antioxidants into 3D-printed things. It shows how methods like Selective Laser Sintering (SLS), Fused Deposition Modeling (FDM), and Stereolithography (SLA) match up with techniques such as mixing antioxidants directly, putting them on the outside, or trapping them inside.

**TABLE 2:** The appropriate incorporation method for each 3D Printing technology based on various supporting references.

3D printing technology	Antioxidant delivery approach	References
Multi-Jet Printing (MJP)	Direct Mixing	(Uddin et al., 2015; Acosta-Vélez et al., 2018; Kollamaram et al., 2018)
Stereolithography (SLA)	Coating	(Economidou et al., 2019; Uddin et al., 2020)
	Direct Mixing	(Sun et al., 2016; Martinez et al., 2018; Karakurt et al., 2020)
Digital Light Processing (DLP)	Direct Mixing	(Lim et al., 2017; Kadry et al., 2019; Krkobabić et al., 2019; Yang et al., 2020)
Fused Deposition Modelling (FDM)	Coating	(Zhou et al., 2018; Stewart et al., 2020)
	Hot melt extrusion	(Goyanes et al., 2016; Skowyra, Pietrzak, and Alhnan, 2015; Tappa et al., 2017; A Melocchi et al., 2019; Mathew et al., 2019)
	Direct mixing	(Persaud et al., 2020)
Pneumatic Extrusion (PE)	Direct mixing	(Ahlfeld et al., 2017; Goyanes et al., 2019; Sjöholm and Sandler, 2019; Li et al., 2020; Ong et al., 2020)
Binder Jetting (BJ)	Direct mixing	(Tian et al., 2018; Acosta-Vélez et al., 2017; Katstra et al., 2000; Wu et al., 2009; W; Huang et al., 2007)
Selective Laser Sintering (SLS)	Direct mixing	(Salmoria, Klauss, and Kanis, 2017; Awad et al., 2019; Trenfield et al., 2020)

## 5 Impact of material properties on antioxidant release kinetics and stability

The selection of materials for 3D printing-assisted antioxidant delivery systems plays a critical role in determining the release kinetics and stability of antioxidants within the printed structures. Material properties such as porosity, degradation rate, hydrophilicity, and compatibility with antioxidants significantly influence the release behavior and long-term stability of the incorporated antioxidants (Azad et al., 2020; Lion et al., 2021). Porosity is an important parameter that affects the diffusion and release kinetics of antioxidants (López de Dicastillo et al., 2011; Chew, Arriaga, and Hinojosa, 2016). Highly porous materials provide a larger surface area for antioxidant release, facilitating faster diffusion and potentially leading to a burst release effect. On the other hand, materials with lower porosity may exhibit slower release profiles, enabling sustained delivery of antioxidants over an extended period. Optimizing the porosity of the printed structures can be achieved through adjusting printing parameters such as infill density or incorporating sacrificial materials that can be removed post-printing. The role of porosity in controlling the diffusion and release kinetics of antioxidants cannot be overstated. For instance, by adjusting the infill density of scaffolds printed using FDM, the porosity of the scaffold can be directly modulated. A higher infill percentage, 80% for instance, creates a more densely packed structure, leading to reduced porosity. Consequently, this might cause slower antioxidant release due to limited diffusion channels. On the other hand, a lower infill density, like 20%, increases porosity, potentially speeding up the diffusion and causing a burst release effect, especially in the initial stages. Moreover, porosity can also be influenced by the choice and handling of materials. For example, the use of sacrificial materials, such as water-soluble PVA (polyvinyl alcohol), can be strategically placed within the scaffold. Post-printing, these can be leached out, leaving behind a porous structure. The advantage here is the ability to achieve specific and intricate pore geometries, which can guide antioxidant release in predetermined patterns. Another technique involves adding porogens, which are materials that induce pore formation. Salt, sugar, or gelatin particles, when mixed with the primary printing material, can act as temporary fillers. Once the printing is done, these particles can be washed away or dissolved, resulting in increased porosity. The size and distribution of the porogen particles directly influence the resultant pore sizes and their interconnectivity. A real-world example is the encapsulation of Vitamin E, a commonly used antioxidant in tissue engineering. When embedded within a highly porous scaffold, its release is more rapid, beneficial for immediate therapeutic needs. In contrast, for scenarios where prolonged, steady antioxidant activity is desired, embedding Vitamin E in low porosity structures would be more appropriate.

The degradation rate of the printing materials also impacts the release kinetics and stability of antioxidants (Xu et al., 2017; Rahmani, Elshereef, and Sheardown, 2021). Materials with faster degradation rates will result in more rapid degradation of the matrix and subsequent release of antioxidants. Conversely, materials with slower degradation rates may provide a longer-term release profile. Careful consideration of the degradation kinetics of the printing materials is necessary to ensure the desired release profiles and the stability of the incorporated antioxidants during the intended application timeframe.

The hydrophilicity of the printing materials influences the interaction between the antioxidants and the surrounding environment (Panraksa et al., 2021). Hydrophilic materials tend to absorb more water, which can accelerate the degradation of the matrix and impact the release kinetics of antioxidants. Conversely, hydrophobic materials may hinder the diffusion of water into the matrix, potentially prolonging the release of antioxidants. The hydrophilicity of the printing materials can be tailored through material selection or post-processing techniques to modulate the release behavior of antioxidants.

Compatibility between the printing materials and antioxidants is crucial for maintaining the stability and functionality of the incorporated antioxidants (Highley et al., 2019; Jongprasitkul et al., 2022). Incompatible materials can lead to chemical reactions, degradation, or loss of antioxidant activity. It is essential to consider the solubility, chemical reactivity, and physical interaction between the antioxidants and the printing materials. Compatibility testing and characterization methods can be employed to assess the stability and compatibility of the selected materials and antioxidants.

Post-processing methods, such as curing, crosslinking, or surface treatments, can also influence the release kinetics and stability of the incorporated antioxidants (Wallner et al., 2022). These methods can further modify the material properties, including porosity, degradation rate, and surface characteristics, thereby impacting the release behavior. Optimization of post-processing methods is necessary to achieve the desired release profiles and enhance the stability of the antioxidant delivery system. Curing is a process where materials undergo a phase transition, usually from a liquid or semi-solid state to a solid state, through the application of heat or light. For example, photopolymer resins in 3D printing often undergo UV curing. If antioxidants are incorporated within these resins, curing can potentially alter their release kinetics. For instance, a more thorough cure might result in a tighter polymer network, which could slow down the diffusion and release of antioxidants. Conversely, under-cured systems might lead to faster release due to looser polymer structures. Crosslinking is the process of forming bonds between polymer chains, enhancing the material’s rigidity. This can be achieved chemically or physically. Hydrogels are a classic example. When used in 3D printing, their crosslinking density can be tuned to control the release of incorporated agents. For instance, a denser crosslinked hydrogel would generally release its payload (like antioxidants) more slowly due to its restricted diffusion channels, while a loosely crosslinked hydrogel may offer a faster release. The surface properties of 3D printed constructs can be altered post-printing through various surface treatments. Plasma treatment, for instance, can introduce certain functional groups onto the surface, which might enhance or restrict the release of antioxidants, depending on the chemistry involved. Another example is the coating of printed structures with bioactive layers, which can act as barriers, modulating the release kinetics of antioxidants. For instance, a printed scaffold coated with a thin layer of chitosan might show a delay in antioxidant release due to the additional barrier the coating presents.

One specific example involves Vitamin C, a potent antioxidant sensitive to environmental conditions. If incorporated within a 3D printed hydrogel, the rate of crosslinking can directly impact its release. A denser crosslinked structure may protect the Vitamin C from rapid degradation and ensure a sustained release, beneficial for applications where prolonged antioxidant activity is desired. Conversely, for immediate therapeutic needs, a loosely crosslinked gel, possibly combined with post-print surface treatments to enhance immediate release, would be more appropriate.

### 5.1 Optimization of printing parameters for achieving desired antioxidant distribution

The distribution of antioxidants within 3D-printed structures is influenced by various printing parameters, which can be optimized to ensure uniform and desired antioxidant distribution. Achieving an even distribution is essential for consistent release kinetics and therapeutic efficacy of the antioxidant delivery system. Several key printing parameters that can be adjusted to optimize antioxidant distribution include layer height, infill density, printing speed, and printing temperature (Khan et al., 2018; Okafor-Muo et al., 2020; Panić et al., 2022).

Layer height, or the thickness of each printed layer, directly impacts the resolution and precision of the printed structures. A smaller layer height allows for finer details and improved accuracy, which can result in better distribution of antioxidants throughout the printed construct. By reducing the layer height, the printing resolution is increased, enabling the incorporation of antioxidants into smaller and more intricate features of the structure.

Infill density refers to the percentage of the internal volume of the printed structure that is filled with material. Higher infill density leads to a more solid and homogeneous structure, promoting better distribution of antioxidants. By increasing the infill density, a higher volume of the printing material is available for incorporating antioxidants, enhancing their distribution throughout the structure. Optimizing the infill density based on the specific requirements of the antioxidant delivery system can improve the overall distribution and release behavior of antioxidants.

Printing speed and temperature also play significant roles in antioxidant distribution. Faster printing speeds can result in insufficient time for the material to melt and blend properly, potentially leading to uneven distribution of antioxidants. Slower printing speeds allow for better control over material deposition and can contribute to improved antioxidant distribution. Similarly, printing temperature affects the material’s viscosity, flowability, and blending characteristics. Optimizing the printing temperature ensures proper material mixing and distribution of antioxidants, avoiding potential clustering or aggregation.

Additionally, the use of support structures during the printing process can impact antioxidant distribution. Support structures are temporary structures used to provide stability for overhanging or complex geometries during printing. They can affect the flow of the printing material and hinder antioxidant distribution in certain areas. Proper placement and design of support structures are crucial to minimize their impact on antioxidant distribution and ensure uniformity throughout the printed structure.

To optimize antioxidant distribution, it is important to conduct systematic experimentation and characterization. Various imaging techniques, such as microscopy or computed tomography (CT) scanning, can be used to assess the distribution and homogeneity of antioxidants within the printed structures. Adjustments to printing parameters can then be made based on the observed distribution patterns to achieve the desired antioxidant distribution.

### 5.2 Evaluation of post-processing techniques for enhancing antioxidant release and bioactivity

Post-processing techniques play a significant role in optimizing the release kinetics and bioactivity of antioxidants within 3D-printed structures. These techniques modify the printed structures through curing (Garcia, Ayranci, and Qureshi, 2020), surface treatments (Liu et al., 2021), or additional processing steps to enhance the performance of the antioxidant delivery system. Evaluation of post-processing techniques is crucial to ensure the desired release profiles and bioactivity of the incorporated antioxidants.

Curing is an employed post-processing technique that improve the structural integrity and stability of the printed structures. This method involves the application of heat, UV light, or chemical agents to induce chemical reactions and strengthen the material matrix. By enhancing the mechanical properties and durability, curing and crosslinking techniques can provide a more robust framework for the controlled release of antioxidants. Evaluation of the post-curing or crosslinking processes includes assessing the mechanical properties, structural stability, and release behavior of the antioxidants.

Surface treatments are another important aspect of post-processing to optimize the release and bioactivity of antioxidants. Surface modifications can alter the surface characteristics of the printed structures, affecting the interaction between the antioxidants and the surrounding environment. Techniques such as plasma treatment, chemical functionalization, or coating deposition can be employed to modify the surface properties, such as hydrophilicity, roughness, or charge, which can impact the release kinetics and bioactivity of antioxidants. Evaluation of surface-treated structures involves characterization of surface properties, release kinetics, and bioactivity assays.

Evaluation of post-processing techniques can be performed through a combination of characterization methods and bioactivity assays. Techniques such as scanning electron microscopy (SEM), Fourier-transform infrared spectroscopy (FTIR), or thermal analysis can be used to assess structural changes, surface modifications, and stability of the printed structures. *In vitro* and *in vivo* studies can be conducted to evaluate the bioactivity, antioxidant efficacy, and therapeutic effects of the released antioxidants.

## 6 Therapeutic 3D printed antioxidants/antioxidants properties

Within this section, an exploration into the versatile realm of 3D Printed antioxidants is made, presenting a unified narrative across four distinct subsections, each highlighting a unique real-world application gleaned from existing literature. These subsections encompass: 1. 3D-printed bone scaffolds, 2. functional antioxidant-infused foods, 3. wound healing with 3D printing, and 4. *in vivo* tissue cultures through 3D Printing.

### 6.1 3D-printed bone scaffolds

3D-printed bone tissue engineering presents an alluring avenue for alternative treatments targeting bone cancer and orthopedic trauma. These 3D-printed bone scaffolds not only address critical-sized defects in cancer patients post-surgery but also enable the creation of patient-specific anatomical implants using cutting-edge 3D printing technology. The inherent characteristics of 3D Printing, such as rapid production, precision, and direct control through computer-aided design (CAD), make it ideal for manufacturing bone tissue scaffolds. Traditionally, calcium phosphate ceramic scaffolds, particularly hydroxyapatite (HA) and β tricalcium phosphate (β-TCP), are utilized as inorganic components due to their osteoinductive, osteoconductive, and biodegradable properties. The porosity within these scaffolds is crucial, influencing not only the growth of new bone but also vascularization by providing space for cell proliferation and nutrient supply.

Although extensive research on 3D-printed bone tissue engineering has been conducted over the past decade, recent reports have highlighted the innovative use of antioxidants in the development of 3D-printed bone tissue scaffolds. For instance, Bose, Sarkar, and Banerjee (2018) introduced a groundbreaking approach with 3D-printed interconnected macroporous β-TCP scaffolds, loaded with curcumin-PCL-PEG for bone regeneration. Curcumin, known for its antioxidant, anti-inflammatory, and anticancer properties, faces limited *in vivo* use due to its hydrophobic nature. Encapsulation within a poly (ε-caprolactone) (PCL)—polyethylene glycol (PEG) polymeric system enhances curcumin’s bioavailability and enables controlled release. *In vitro* assays demonstrated that the curcumin-PCL-PEG system supports osteoblast proliferation on HA plasma-coated Ti6A14v samples. Subsequent *in vivo* experiments using cylindrical scaffolds (3.2 mm diameter, 5 mm height, and 400 µm pores) revealed that curcumin-coated bone-like scaffolds significantly enhanced bone formation (from 29.6% to 44.9%) compared to control TCP scaffolds in rats. This curcumin-loaded scaffold exhibited remarkable wound healing and osteogenesis capabilities, presenting a novel multifunctional bone tissue engineering scaffold. Beyond targeting cancer cells, it promotes bone formation within the porous scaffold, showcasing potential as a promising drug delivery strategy to address bone defects post-tumor resection. This work was further extended by Sarkar and Bose (2019) to fabricate 3D-printed bone tissue cylindrical scaffolds featuring interconnected porosity, loaded with liposome-encapsulated curcumin. This approach further elevates curcumin’s bioavailability. Constructed from tricalcium phosphate (TCP) ceramic, known for stimulating bone formation and repair, these scaffolds exhibited heightened viability of healthy bone cells (osteoblasts) while inhibiting proliferation of bone cancer cells (osteosarcoma) *in vitro*. Upon implantation at femoral defects in rats, this liposomal curcumin-loaded 3D printed porous scaffold effectively promoted bone formation after 6 weeks, demonstrating its potential as a novel bone graft substitute for regenerative purposes following surgical tumor resection.

M. Zhang, Zhai, et al. (2023) introduced a versatile therapeutic approach by integrating cerium oxide nanoparticles (CeO2 NPs) into bioactive glass (BG) to construct 3D-printed scaffolds. These scaffolds create a sequential therapeutic impact, first mitigating inflammation and subsequently promoting osteogenesis to address bone defects. The antioxidative prowess of CeO2 NPs effectively combats oxidative stress during bone defect formation. Moreover, CeO2 NPs bolster the growth and osteogenic differentiation of rat osteoblasts, enhancing mineral deposition, alkaline phosphatase activity, and the expression of osteogenic genes. The inclusion of CeO2 NPs significantly enhanced the mechanical properties, biocompatibility, cell adhesion, osteogenic potential, and multifunctionality of BG scaffolds within a singular platform. This was validated by *in vivo* experiments on rat tibial defects to prove the superior osteogenic properties of CeO2–BG scaffolds over pure BG scaffolds.

### 6.2 Functional foods with 3D printed antioxidants

In recent years, an escalating focus among consumers on the intrinsic link between health and nutrition has emerged as a novel trend. This trend primarily revolves around the integration of natural elements into food products. S.F. Vieira, Ferreira, and Neves (2020) conducted a study to enhance the antioxidant properties of 3D printed cookies by fortifying them with the microalga Arthrospira platensis. The antioxidants from A. platensis were extracted using ultrasound-assisted methods in hydroalcoholic solutions. Optimization of the ethanol/water ratios and biomass/solvent ratios was carried out using a Design of Experiments (DOE) approach, with antioxidant activity (measured through ORAC and ABTS assays) and total phenolic content (TPC) as the response variables. The most potent values for ORAC, ABTS, and TPC were obtained from an extract containing 0% ethanol and 2.0% biomass. This specific extract was selected for incorporation into a printable cookie dough. The cookies possessed sufficiently low water activity (aw) levels to ensure microbiological stability, and their texture remained consistent even after 30 days of storage. Furthermore, encapsulating the extract led to a notable enhancement in ORAC values and color stability, outperforming other formulations.

**FIGURE 5:**
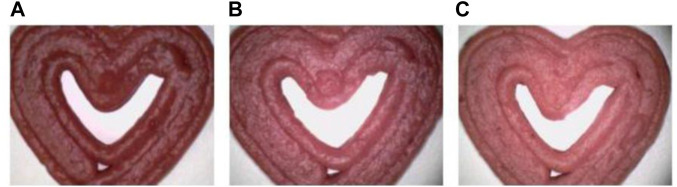
Microscopic images (at ×500 magnification) of 3D printed samples: Impact of incorporated starch levels, 10% **(A)**, 15% **(B)**, and 20% **(C)**, on the degree of lightness (L*). Image from Bebek Markovinović et al. (2023) with permission.

Similarly, Bebek Markovinović et al. (2023) conducted a study aimed to harness 3D Printing for creating a functional strawberry-based product by incorporating two hydrocolloids (corn and wheat starch) in varying proportions (10%, 15%, and 20%), see Figure 5. The research also explored the impact of 3D Printing process parameters on physio-chemical attributes, textural qualities, bioactive and antioxidant potential, and microbiological stability. Additionally, the study investigated the effects of incorporating natural antimicrobial agents on these parameters. The type of starch used exerted a noteworthy influence on all assessed bioactive compounds and starch content, except for total phenolic and hydroxycinnamic acid contents. Remarkably, all samples exhibited favorable textural properties, demonstrated substantial stability, and showed minimal geometric discrepancies.

### 6.3 Wound healing

**FIGURE 6:**
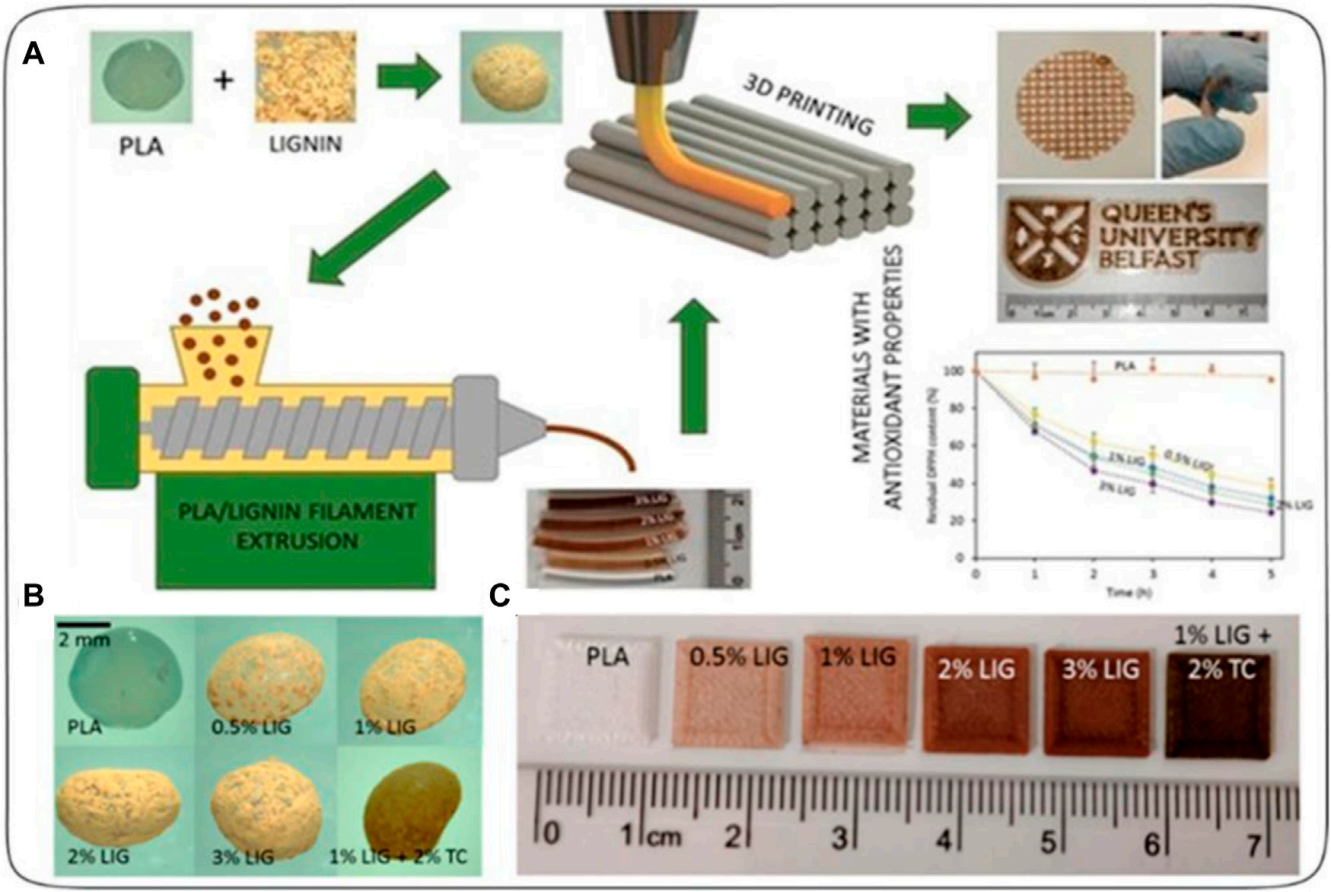
**(A)** Schematic representation of the experimental setup to prepare antioxidant materials used for 3D printed meshes. **(B)** Photographs of poly(lactic) acid (PLA) and PLA-coated pellets. **(C)** Lignin (LIG) and tetracycline (TC) containing squares fabricated using 3D Printing [reprinted from (Domínguez-Robles et al., 2019) with permission].

Domínguez-Robles et al. (2019) created a 3D printed antioxidant material for wound healing see Figure 6. Lignin (LIG), an antioxidant biopolymer, was combined with poly(lactic acid) (PLA) pellets using hot-melt extrusion and castor oil. Antibiotic tetracycline (TC) was also added to prevent bacterial infection. The LIG/TC-coated PLA pellets were utilized to print different shapes via fused filament fabrication (FFF). The printed materials exhibited higher antioxidant activity and antimicrobial properties compared to controls. A two-layered PLA/LIG mesh, along with a polyvinyl alcohol (PVA) film, displayed delayed curcumin release, making it suitable for chronic wound care. This innovative antioxidant PLA/LIG composite holds promise for healthcare applications, with FFF’s versatility enabling tailored designs for patient-specific needs, such as customized wound dressings and scaffolds.

In another study, Tiboni et al. (2020) devised a tailored 3D-printed microfluidic chip to create ethanolic liposomes loaded with glycyrrhetinic acid (GA). GA, a hydrophobic triterpene saponin, showcases anti-inflammatory and antioxidant effects when applied topically. To enhance its bioavailability, GA-loaded liposomes were formulated, incorporating ethanol as a skin permeation enhancer. A biodegradable PLA microfluidic chip was 3D-printed using Fused Deposition Modeling (FDM) for efficient liposomal dispersion. The optimal formulation displayed enhanced efficiency and stability, outperforming free GA. The liposomal hydrogel exhibited superior drug release and permeation compared to control formulations, indicating its potential as a controlled release system for hydrophobic bioactives. This technique offers a cost-effective approach for topical dosage forms.

### 6.4 Tissue *in vivo* 3D cell cultures

3D cell culture models offer numerous advantages over traditional 2D cultures. By cultivating cells in a 3D environment, the natural anatomy and physiology of tissues are replicated, resulting in cell shapes (ellipsoid/polarized) that mirror those found in cell-to-cell junctions and create a heterogeneous cell interface with the medium. These conditions play a role in influencing essential cellular processes, including differentiation, metabolism, gene expression, and morphogenesis. Consequently, the development of an advanced 3D culture system has become essential across diverse domains such as drug discovery and tissue engineering.

A wide array of materials has been utilized for crafting 3D cell cultures. Notably, antioxidants are particularly attractive for such cultures due to their ability to scavenge free radicals and their supplementary advantages, including anti-inflammatory effects. A recent innovation introduced a novel approach to address H2O2-induced oxidative stress and promote robust cell growth and angiogenesis. Rijal et al. (2017) pioneered the development of a large-sized 3D scaffold that entraps catalase to counteract hydrogen peroxide (H2O2) damage during tissue regeneration. Excessive H2O2 accumulation from cells poses a significant challenge in successful large tissue-engineered grafting. The catalase-coated gel, composed of alginate and decellularized adipose tissue matrix (DAT) hydrogel, was applied to a 3D printed polycaprolactone (PCL) scaffold with specific dimensions. The catalase enzymatically detoxified H2O2, generating oxygen and water as byproducts. This prevented the microenvironment from becoming hypoxic and promoted cell survival. Evaluation of gel stability within the coated PCL scaffolds showcased enhanced performance with DAT-alginate gel, which released catalase in a sustained manner. *In vitro* findings revealed that catalase release from the 3D scaffold effectively protected human turbinate mesenchymal stem cells (hTMSCs) from oxidative stress induced by H2O2. An *in vivo* study involving subcutaneous-implanted scaffolds in rats exhibited reduced inflammation (≥40%), increased tissue growth (≥45%), and induction of angiogenesis (≥40%). This innovative model holds great promise for regenerating damaged tissues.

Additionally, X. Zhang et al. (2020) introduced a 3D-printed cellulose nanofibril-alginate-spherical colloidal lignin particle (CNF-alginate-CLP) nanocomposite scaffold for three-dimensional cell culture. The use of cellulose nanofibril (CNF) hydrogels, known for their hydration capacity, biocompatibility, and shear-thinning properties, has gained attention in 3D printing applications for cell cultures and tissue engineering. The incorporation of spherical lignin nanoparticles not only provided antioxidant properties but also enhanced printing resolution and shape stability through viscosity modulation. The 3D bioprinting process involved the use of biomaterial inks comprising CNF hydrogel, alginate, and CLP dispersion on polypropylene Petri dishes. The resulting CNF-alginate-CLP scaffolds maintained shape stability during storage and demonstrated consistent proliferation of cells. This suggests significant potential for the CLP-containing scaffold in soft-tissue engineering applications within the realm of regenerative medicine.

## 7 Challenges and future directions

While 3D-printed antioxidant systems hold great promise in various applications, there are several challenges that need to be addressed to fully harness their potential, apart from the challenges associated with 3D Printing (e.g., Abdulhameed et al., 2019; A; Alogla, Baumers, and Tuck, 2021). One of the key challenges is the selection and optimization of antioxidant materials. Identifying antioxidants with the desired stability, bioactivity, and controlled release properties is crucial for achieving effective therapeutic outcomes. Further research is needed to explore novel antioxidant candidates and develop methods for their efficient incorporation into 3D-printed structures.

Another challenge lies in the precise control of antioxidant release kinetics. Achieving the desired release profiles, whether it is sustained release, pulsatile release, or triggered release, requires a deep understanding of the material properties, printing parameters, and formulation strategies. Developing sophisticated release mechanisms and designing constructs that provide tailored release kinetics will be critical for optimizing the therapeutic effects of 3D-printed antioxidant systems. Additionally, the regulatory landscape surrounding 3D-printed medical devices and drug delivery systems needs to be further established. As the field progresses, it is important to ensure that these technologies meet the necessary safety and efficacy standards. Regulatory bodies and industry stakeholders need to collaborate to develop guidelines and standards that facilitate the translation of 3D-printed antioxidant systems from the laboratory to clinical practice.

Furthermore, scalability and manufacturing efficiency are important considerations for the widespread adoption of 3D-printed antioxidant systems. The fabrication processes need to be optimized to achieve high throughput and cost-effective production without compromising the quality and performance of the constructs. Advances in 3D printing technologies, such as faster printing speeds, improved material formulations, and automation, will play a crucial role in addressing these challenges. Looking ahead, the future perspectives of 3D-printed antioxidant systems are promising. Advancements in materials science, biofabrication techniques, and additive manufacturing technologies will continue to drive innovation in this field. The development of novel antioxidant materials with improved bioactivity and controlled release properties will expand the range of therapeutic applications. Integration with other emerging technologies, such as nanotechnology, bioinks, or bioprinting, may further enhance the functionalities and capabilities of 3D-printed antioxidant systems.

Additionally, the combination of 3D printing with other therapeutic modalities, such as gene therapy or immunotherapy, holds potential for synergistic effects in combating oxidative stress-related diseases. The ability to precisely deliver antioxidants alongside other therapeutic agents may revolutionize treatment strategies and improve patient outcomes. Moreover, the integration of artificial intelligence (AI) and machine learning algorithms with 3D-printed antioxidant systems can facilitate personalized medicine approaches. AI algorithms can assist in designing patient-specific constructs, predicting optimal antioxidant release profiles, and optimizing treatment protocols based on patient-specific data.

## 8 Conclusion

In conclusion, 3D printing technology has revolutionized the field of antioxidant delivery systems, offering innovative approaches for various applications. The ability to incorporate antioxidants within 3D-printed structures provides opportunities for targeted delivery, controlled release, and customization of antioxidant therapies. These systems have shown great potential in applications such as wound healing, tissue regeneration, drug delivery, personalized medicine, and more.

The research and development in 3D-printed antioxidant systems have presented numerous advantages and possibilities. The direct mixing approach allows for the incorporation of antioxidants within the printing materials, ensuring homogenous distribution throughout the constructs. Coating techniques offer surface modification and encapsulation of antioxidants, protecting them from degradation and facilitating controlled release. Encapsulation methods provide strategies for embedding antioxidants within 3D-printed matrices, enhancing their stability and release kinetics. Each approach has its own advantages and limitations, and the selection of the most suitable method depends on the specific requirements of the intended application. Factors such as material properties, printing parameters, and post-processing techniques influence the antioxidant release kinetics and stability. Optimization of these parameters is crucial to achieve the desired therapeutic outcomes.

Furthermore, the development of 3D-printed antioxidant systems faces challenges that need to be addressed. Selection and optimization of antioxidant materials, precise control of release kinetics, regulatory considerations, scalability, and manufacturing efficiency are among the key challenges that require further research and development efforts. Overcoming these challenges will pave the way for the widespread adoption of 3D-printed antioxidant systems in clinical practice. Looking ahead, the future of 3D-printed antioxidant systems is promising. Advancements in materials science, biofabrication techniques, additive manufacturing technologies, and integration with other therapeutic modalities will continue to drive innovation in this field. The combination of 3D printing with other emerging technologies, such as nanotechnology and AI, holds great potential for further enhancing the functionalities and capabilities of antioxidant delivery systems.
